# Re-Centering Clinical Documentation in the Age of AI Scribes: Four Aims of the Patient Chart Note

**DOI:** 10.2196/95218

**Published:** 2026-06-08

**Authors:** Chwen-Yuen Angie Chen, Tiffany I Leung, Rika Bajra

**Affiliations:** 1 Division of Primary Care Population Health, Department of Medicine, Stanford University, 211 Quarry Road, 4th Floor, Palo Alto, CA, 94304, United States, 1 650-498-9000, 1 888 755 4489; 2 JMIR Publications, Toronto, ON, Canada; 3 Department of Internal Medicine, Southern Illinois University School of Medicine, Springfield, IL, United States

**Keywords:** medical education, clinical training, artificial intelligence, ambient scribe, medical documentation

## Abstract

Clinical documentation is a foundational skill in medicine, developed during training and required in everyday practice. Historically, the chart note functioned as a clinician-centered cognitive tool for reasoning, teaching, and communication but has evolved into a multipurpose document shaped by administrative, regulatory, and financial demands, and is increasingly experienced as burdensome. The electronic health record, intended to improve efficiency, has introduced additional complexity and workflow strain, contributing to clinician burnout. Ambient artificial intelligence (AI) scribe technologies are rapidly being adopted to address these challenges, yet their implementation has outpaced evidence regarding their impact on learning, cognition, and clinical reasoning. We raise questions regarding the underexplored consequences of AI-assisted documentation, particularly cognitive off-loading and the potential for de-skilling, echoing historical concerns surrounding earlier cognitive technologies that externalized thought. We propose a practical framework that re-centers clinical documentation around four core aims: supporting clinical reasoning (“note to self”), facilitating communication (“note to others”), meeting medicolegal and billing requirements, and enhancing patient education in the era of open notes. Incorporating this framework into training may promote more intentional documentation practices before routine reliance on AI. We advocate for reframing the chart note to support clinician development and preserve its role in high-quality, patient-centered care.

Clinical documentation has evolved beyond its original, singular purpose of supporting patient care. As health care systems expanded to include the goals of prevention, population health, and data portability, documentation requirements grew in scope and complexity [1]. Secondary uses of the electronic health record (EHR) data, including billing, quality reporting, and regulatory compliance, have further shaped documentation practices and system design [2,3]. What was intended to improve efficiency and care coordination also introduced a significant administrative burden and workflow strain, contributing to clinician burnout and lost revenue from unclosed charts [4-6]. In their systematic review, using a rigorous search strategy across multiple databases assessing EHR documentation, Colicchio and Cimino [7] elaborate on how EHR documentation now supports multiple overlapping and at times competing purposes that have transformed clinical chart notes into a multipurpose artifact, often diluting its primary role as a tool for clinical reasoning and communication. Their review suggests that EHR documentation often fails to adequately capture clinicians’ integrated understanding of the patient: how presentation, problems, interventions, and goals are meaningfully connected as well as what clinicians are “thinking” about the patients’ problems.

Considering these challenges, a re-centering of the purpose of clinical documentation is needed. In this context, we propose four core aims of the clinical chart note as a pragmatic, clinician-centered framework that selectively prioritizes key functions most essential to patient care and professional development. This “four aims” framework can be viewed as a normative, pedagogical approach designed to help clinicians and trainees navigate these tensions of overlapping purposes and increasing external pressures of clinical charting, while reflecting both the historical functions and enduring purpose of clinical documentation, especially in the era of artificial intelligence (AI)–assisted ambient documentation. The work of Colicchio and Cimino [7] also invites renewed focus on the most used section of the note—the assessment and plan—as an opportunity to clearly articulate clinical reasoning and synthesize the patient’s situation, including social determinants of health, in a language accessible to both clinicians and patients.

The four aims are ordered to mirror the historical development of medical documentation. The first two purposes, delivering good patient care, remain unchanged; the third purpose arose with malpractice liability; and as open notes have become widespread, the fourth purpose of patient education has evolved:

1. Reminder to self: The chart note serves as a reminder of clinical reasoning, salient findings, and interventions, supporting continuity of care.
2. Communication with other professionals: Documentation communicates pertinent information and rationale to colleagues for care continuity.
3. Medicolegal and administrative record: Accurate documentation supports legal protection, quality control, and financial requirements; activity that one forgets to document essentially did not occur, and activity that did not occur should not be carried over in a note.
4. Patient education: Sharing information in lay terms educates patients and their caregivers; open notes support transparency and ownership of one’s own health information and allow patients into clinical reasoning.

Examining the history of clinical documentation reveals that the burden of charting is not inherent to the act itself, but rather a consequence of how its purpose has expanded over time to accommodate administrative, regulatory, and financial demands. Understanding this evolution helps contextualize modern burnout and creates space to re-center documentation around its most meaningful functions. Looking back, medical documentation is as old as cave illustrations of injury, healing rituals, and death [8]. With the advent of written records, ancient civilizations documented illness and treatment to transfer knowledge across generations. Over time, these ancient scripts evolved from narrative accounts into more structured approaches to catalogs of patient cases for education and communication [8]. In 1968, Dr Lawrence Weed introduced the subjective, objective, assessment, and plan (SOAP) note format [9], which was followed by the problem-oriented medical record (POMR) [10] and later adaptations such as the assessment, plan, subjective, and objective (APSO) format [11]. These frameworks were eventually embedded within the EHR.

The rise of human medical scribes over the past decade reflects an effort to off-load documentation tasks in response to increasing administrative burden [12]. However, the impact of scribes, whether human or AI, on documentation quality and clinician workload remains mixed [13]. Clinicians’ individual narrative styles may support recall and understanding, including an emotional connection with the patient, thus creating a cognitive imprint that may be diminished when documentation is delegated. Yet the widespread and rapid adoption of AI-assisted documentation has outpaced evidence regarding its effect on cognition and clinical reasoning [14,15]. Emerging evidence suggests that the use of large language models in writing tasks may reduce memory recall, perceived ownership, and the ability to reproduce one’s own work [16]. More importantly, it is now widely discussed how reliance on AI scribes introduces cognitive off-loading that may reduce clinicians’ active engagement in synthesizing and documenting clinical encounters, processes essential to critical thinking and clinical reasoning [17,18].

Concerns about cognitive off-loading are not new. Early critics of writing warned that externalizing knowledge would weaken memory and internal understanding [8]. The evolution of documentation reflects an ongoing balance between cognitive work, efficiency, and standardization. Early narrative records were an expression of the individual clinician’s interpretation and memory of a clinical encounter or experience, while the introduction of structured formats such as SOAP and POMR reflected a shift toward formalizing clinical reasoning for teaching and reproducibility. Each transition introduced trade-offs: increased standardization improved communication and scalability but risked constraining narrative nuance and individual cognitive processes. Educators adapted by incorporating these formats into training, using documentation as a tool to externalize and teach clinical reasoning. Dictation devices, EHR templates, copy-and-paste practices, and now AI-assisted documentation all represent a continuation of these shifts. However, unlike prior changes, AI introduces the possibility of not only delegating documentation tasks but also off-loading the process of cognitive work itself. This distinction raises important questions about how current innovations compare with prior disruptions and whether they may fundamentally alter the role of documentation as a tool to teach clinical reasoning in medical education.

Educators have raised concerns that cognitive off-loading from AI tools may diminish the “desirable difficulties” necessary for developing deep understanding and critical thinking [19-21]. Historically, trainees have grappled with writing patient encounter notes, first by hand and later digitally, a productive struggle that supports the development of essential clinical reasoning skills. Although the longitudinal impact of ambient AI scribes remains unclear, educators caution that introducing these tools before trainees establish foundational competencies may lead to “never-skilling,” in which essential skills fail to develop due to premature cognitive off-loading [17,18,22]. Even for advanced learners, passive reliance on AI-generated drafts risks automation bias [17], allowing errors or embedded biases to go unrecognized and potentially negatively impacting patient-provider relationships and the quality of care. A competency-based approach [23-27] would introduce ambient AI scribes only after learners demonstrate readiness through observable documentation behaviors, supported by entrustment decisions indicating their ability to independently achieve the four aims of clinical documentation. This transition should be scaffolded by a longitudinal AI curriculum that assesses competencies in critical appraisal of AI-generated notes (identifying errors of omissions, hallucinations, and inaccuracies) [28-31], bias recognition and mitigation (eg, stereotyped language or speech recognition errors in patients with limited English proficiency) [32-39], and independent verification of the accuracy of AI-assisted notes as a core component of professional accountability. Drawing on their internal medicine residency pilot at Johns Hopkins, Abernethy et al [27] advocate that residents demonstrate foundational note-writing competence before using AI scribes and that residents receive structured AI teaching to monitor for hallucinations and recognize algorithmic bias that may be embedded in the training data, including stigmatizing language, misattribution of pronouns, and omission of social determinants of health [27]. Educational strategies could include the discussion, evidence, feedback, and training (DEFT) AI framework, applied during clinical supervision to explore trainees’ clinical reasoning and reinforce a “verify, then trust” approach that promotes vigilance against overreliance and automation bias, while applying the four aims framework.

While we are optimistic about the tangible benefits of ambient AI scribe use, we remain concerned that its implementation is outpacing robust evaluation of its impact on the cognitive development and practices of physicians, trainees, and other health professionals. Do we have the skills to discern accurate and safe use of ambient AI scribes? What are the ramifications of patients verifying the accuracy of our open documentation, especially if we are not in the habit of reviewing AI-scribed notes? Current evaluations of ambient AI scribes primarily focus on note quality, productivity, burnout, and user satisfaction, but have yet to adequately address these other questions. AI will inevitably bring new ways of learning and integrating knowledge, just as past advances have done. In the meantime, we advocate that the four main aims of the chart note, whether handwritten or digitized, serve as practical guidance and a rubric for trainee feedback regardless of what is gained and lost in this evolution of medical knowledge processing.

Ultimately, the essence of the chart note is to preserve accuracy, communicate with clarity, provide transparency, maintain patient dignity, and most importantly, exhibit that which is innately human: our personal connections to each other.

## Abbreviations

AI: artificial intelligence
APSO: assessment, plan, subjective, and objective
DEFT: discussion, evidence, feedback, and training
EHR: electronic health record
POMR: problem-oriented medical record
SOAP: subjective, objective, assessment, and plan
